# What makes a good prediction? Feature importance and beginning to open the black box of machine learning in genetics

**DOI:** 10.1007/s00439-021-02402-z

**Published:** 2021-12-04

**Authors:** Anthony M. Musolf, Emily R. Holzinger, James D. Malley, Joan E. Bailey-Wilson

**Affiliations:** 1 Statistical Genetics Section, Computational and Statistical Genomics Branch, National Human Genome Research Institute, National Institutes of Health, 333 Cassell Drive Suite 1200, Baltimore, MD 21224 USA; 2 Target Sciences, Informatics and Predictive Sciences, Bristol Myers Squibb, Cambridge, MA USA

## Abstract

Genetic data have become increasingly complex within the past decade, leading researchers to pursue increasingly complex questions, such as those involving epistatic interactions and protein prediction. Traditional methods are ill-suited to answer these questions, but machine learning (ML) techniques offer an alternative solution. ML algorithms are commonly used in genetics to predict or classify subjects, but some methods evaluate which features (variables) are responsible for creating a good prediction; this is called feature importance. This is critical in genetics, as researchers are often interested in which features (e.g., SNP genotype or environmental exposure) are responsible for a good prediction. This allows for the deeper analysis beyond simple prediction, including the determination of risk factors associated with a given phenotype. Feature importance further permits the researcher to peer inside the black box of many ML algorithms to see how they work and which features are critical in informing a good prediction. This review focuses on ML methods that provide feature importance metrics for the analysis of genetic data. Five major categories of ML algorithms: k nearest neighbors, artificial neural networks, deep learning, support vector machines, and random forests are described. The review ends with a discussion of how to choose the best machine for a data set. This review will be particularly useful for genetic researchers looking to use ML methods to answer questions beyond basic prediction and classification.

## Introduction

In the past decade, genetic data have become more and more complex. Researchers now have easy access to whole-genome sequence (WGS), whole-exome sequence (WES), RNA sequencing (RNA-seq), and other forms of gene expression and proteomics data. As the data size and complexity has increased, the questions geneticists have begun to answer have increased at a commensurate rate. For example, researchers now want to know whether disease risk is caused by epistatic (gene–gene) interactions between germline genetic variants or what effect a single missense variant in the DNA might have on the structure, and consequently the function, of a protein. With traditional methods often struggling to provide sufficient answers to these questions, geneticists have begun to frequently turn to machine learning (ML) techniques (Moore et al. 2010; Moore and Williams 2009).

ML methods can be broadly defined as the algorithms (machines) that can learn from data to make predictions or identify patterns too complex for humans to detect. ML uses a sample set of data, called a training set, to initially program or construct the prediction model. Further input into the machine will then give an output based on how the machine was trained. ML is considered a branch of artificial intelligence (AI).

There are multiple types of ML techniques, many of which have been adapted to genetic data. The vast majority of ML algorithms in genetic analysis are used in prediction, essentially using some input data to predict an outcome or the value of a trait for a particular subject. In genetic data, this could be using SNP genotypes to predict whether a subject is a case or control or using gene expression data to predict whether a tumor is likely to metastasize.

Good predictions are indeed critical in genetics. However, identifying which variables (in ML parlance called features) are most responsible for informing these good predictions is also critical. The difference between prediction, estimation, and attribution has been addressed by many authors, and is well described by, for example, Efron (2020), Malley et al. (2011), and Hastie et al. (2009). ML algorithms, especially the more sophisticated ones, seem like black boxes. You put in some data and get out a prediction. However, in genetics, we not only want to get a good prediction—we want to know which variables, such as age, sex, environmental exposures, and genetic variant genotypes, are important at predicting disease or predicting which genes are differentially expressed in metastatic versus benign tumors.

This is called feature importance in ML—knowing which features are the best predictors. Feature importance (which is also called feature detection, feature attribution, or model interpretability and is related to the statistical ideas of estimation and attribution) will output a particular score or metric, permitting ranking of features from largest to smallest contribution to the machine’s prediction. They are often obtained by systematically permuting features, to determine which feature causes the largest change in predictive power. This will produce an importance score for each feature, allowing for feature ranking. For example, this would allow researchers to identify which genetic variants might be associated with disease and then earmark them for functional studies. It would also allow a protein prediction program to identify which set of input data were most important at creating a good prediction. Thus, it is important to keep in mind while reading this manuscript that different feature importance metrics measure different things and are thus not comparable; indeed, many importance measures are not interpretable in general.

This review sets out to outline some of the different software programs that provide feature importance metrics using genetic data; it is not meant to be an exhaustive review of the subject. For the scope of this paper, genetic data will be defined as the analysis of DNA or RNA sequence data. Much of this paper will concern two major topics. The first is determining which genetic variants are associated with a given disease, specifically which variants increase the risk of disease. Here, the predictor variable will be variant genotypes and the dependent variable will be disease status, which can be binary or quantitative. Thousands of variant genotypes (millions with whole-genome sequence data) are analyzed and typically subjects number in the hundreds or thousands, dependent on the prevalence of the disease being studied. An important offshoot of this topic is finding epistatic interactions between variants, i.e., which variants interact to form an increased disease risk. The second topic we will address is variant pathogenicity. Here, changes in the DNA are extrapolated to protein structures, where one will attempt to predict the damage to the protein structure based on the amino acid substitution caused by the DNA mutation. Here, the independent variable is the variant genotype, while the dependent variables are the amino acid changes and its subsequent effect on the protein. However, we also touch on other uses of machine learning such as approaches that involve RNA sequence data for gene expression analyses.

Feature importance will be discussed across five of the most popular machines—*k* nearest neighbors, artificial neural networks, deep learning, support vector machines, and random forest—before discussing some approaches for choosing the best machine for a particular data set as well as tuning machine parameters. A tabular form of the software discussed is provided in Table 1.

**Table 1:** List of machine learning software

Software name	Machine type	Software type	Application	Data type	Website
SURF	*k*-NN	Part of open-source package	Epistatic interactions	SNP	http://www.epistasis.org
STIR	*k*-NN	Standalone Program	Epistatic interactions	SNP	http://insilico.utulsa.edu/software/STIR
ReliefSeq	*k*-NN	Standalone Program	Epistatic interactions	RNA-seq	http://insilico.utulsa.edu/ReliefSeq.php
KNN-MDR	*k*-NN	Standalone program	Epistatic interactions	SNP	n/a
GANN	ANN	Standalone program	Gene-based expression association	RNA-seq	n/a
ANNI	ANN	Standalone program	Epistatic interactions	SNP	n/a
ATHENA	ANN	Standalone program	Epistatic interactions	SNP	https://ritchielab.org/software/athena-downloads
Basset	Deep learning	Standalone program	Noncoding annotation	DNA-seq	https://github.com/davek44/Basset
DeepSEA	Deep learning	Standalone program	Noncoding annotation	DNA-seq	http://deepsea.princeton.edu/
DeepWAS	Deep learning	Standalone program	GWAS/annotation integration	GWAS	https://github.com/cellmapslab/DeepWAS
DFIM	Deep learning	Standalone program	Epistatic interactions	DNA-seq	https://github.com/kundajelab/dfim
PrimateAI	Deep learning	Standalone program	Variant pathogenicity	DNA-seq	https://basespace.illumina.com
CADD	SVM	Standalone program	Variant pathogenicity	DNA-seq	https://cadd.gs.washington.edu
MSIpred	SVM	Python package	Microsatellite instability prediction	WES	https://github.com/bioinfolabmu/MSIpred
REVEL	RF	Standalone program	Variant pathogenicity	DNA-seq	https://sites.google.com/site/revelgenomics/
Random jungle	RF	R package	GWAS	SNP	https://r-forge.r-project.org/R/?group_id=741
Ranger	RF	R package	GWAS	SNP	https://cran.r-project.org/web/packages/ranger/index.html
Open target genetics	RF	Standalone program	SNP/gene prioritization	GWAS Results	https://genetics.opentargets.org
Permuted RF	RF	Standalone program	Epistatic interactions	SNP	n/a
RF fishing	RF	Standalone program	Epistatic interactions	SNP	n/a
SWSFS	RF	Standalone program	Epistatic interactions	SNP	n/a
r2VIM	RF	Standalone program	Epistatic interactions	SNP	https://research.nhgri.nih.gov/software/r2VIM/
Boruta	RF	R package	Epistatic interactions	SNP	https://cran.r-project.org/web/packages/Boruta/index.html
Vita	RF	R package	Epistatic interactions	SNP	https://cran.r-project.org/web/packages/vita/index.html

A list of the software referenced in this review. The columns represent the software names, the type of machine used in the software, the type of software (i.e., whether the software is a stand-alone program or a package), the application for the software, the type of data the software analyzes (note that programs that use SNP data can also use DNA-seq), and the link to download the software (if available)

## Methods

### K nearest neighbors

**Fig. 1:**
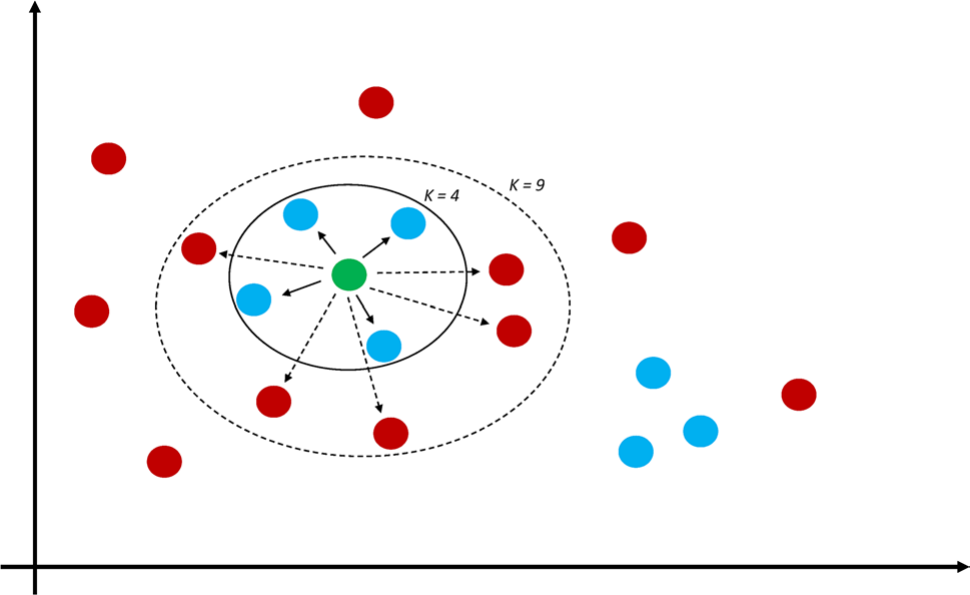
*k*-nearest neighbors. A diagram showing an example of the k-nearest neighbor machine. Subjects are plotted based on feature values, and an individual’s classification is determined by a majority vote in the subject’s neighborhood (*k*). The choosing of *k* is crucial to classification. For instance, if we wished to classify the green individual based on *k* = 4, the individual would be classified as blue. If we extended this to *k* = 9, the individual would be classified as red

*K* nearest neighbors (*k*-NN) is one of the simplest and oldest ML algorithms. However, it can still be as effective as some of the newer, more complex machines (Malley et al. 2011). The basic assumption that underlies the *k*-NN approach is that the classification of a subject into a group (such as case or control in a genetic study) should depend primarily on the other subjects closest to it, its “neighborhood”. While the term neighborhood sounds somewhat nebulous, it is determined by a distance metric between subjects, with the Euclidean distance being a popular choice (Fig. 1). In practice, this works by plotting all subjects in space based on a set of features (e.g., SNP genotypes). For each subject, the machine looks at a predetermined number (termed k) of neighbors with the shortest distance from the subject and a majority vote of these neighbors then determines the subject’s classification. *k*-NN can also be used as a regression analysis for quantitative traits (Devroye et al. 1994).

*k*-NN has been shown to be effective despite its simplicity and has been effectively used in genetic studies (Malley et al. 2011). In genetics, other distances besides Euclidean are often used, such as Mahalanobis distance, which allows for effects like correlation (critical for linkage disequilibrium between variants) (Abo Alchamlat and Farnir 2017) and Hamming distance, which is useful for strings of data (like DNA sequence). For genetic data, identity-by-state (IBS) and identity-by-descent (IBD) distance can also be used; both NetView (Neuditschko et al. 2012) and FastSMC (Nait Saada et al. 2020) use IBD values to determine fine-scale population structures.

Despite its effectiveness, *k*-NN has some major drawbacks. First, the machine requires certain input parameters to run, specifically the choice of k and the type of distance measurement (Abu Alfeilat et al. 2019). Further *k*-NN is computationally intensive, as the distance between all subjects needs to be calculated (Malley et al. 2011). To combat this, filter methods are often applied to decrease the total number of features used in *k*-NN analyses. This could mean using other ML methods to reduce feature input or using annotation, for instance biological function, to whittle down the total number of features.

Feature importance can be determined in *k*-NN. Relief algorithms have overcome the dimensionality issues using wrappers and filters to decrease computation time (Greene et al. 2009; Moore et al. 2006), allowing for assessment of feature importance. Filters rank variables based on a metric that estimates the association with the outcome, independently of the ML method. In contrast, wrappers use the ML algorithm on subsets of the variables to select an optimal set. This has been particularly useful in the study of epistasis in GWAS or sequence data. Spatially Uniform Relief (SURF) assigns each feature a score and has been found to effectively identify pairwise interacting SNPs, even when those SNPs do not have main effects (Greene et al. 2009). While SURF cannot identify the nature of the interaction between the SNPs, it can filter the features based on a Relief score threshold to a number feasible for traditional combinatorial approaches. This list of important features can be used as input to other approaches that can model interactions. A more recent update of the SURF approach, called statistical inference relief (STIR), was developed to calculate feature (attribute) scores without the need for permutation (Le et al. 2019) and was successfully applied to RNA-Seq data. Other genetic uses of Relief algorithms include ReliefSeq, which has been adapted as a gene-based test to find both main effects and gene–gene interactions in mRNA-seq expression data (McKinney et al. 2013).

The KNN-MDR approach (Abo Alchamlat and Farnir 2017) combines *k*-NN with multifactor dimensionality reduction (MDR). This approach substitutes the majority vote of MDR amongst individuals sharing the same genotypes with a majority vote of the *k*-NN of a subject. *P* values are produced for each SNP pair, and the method has > 70% power to detect simulated disease SNPs under two-way and three-way interaction scenarios in simulations with 500 cases and 500 controls.

### Random forest

**Fig. 2:**
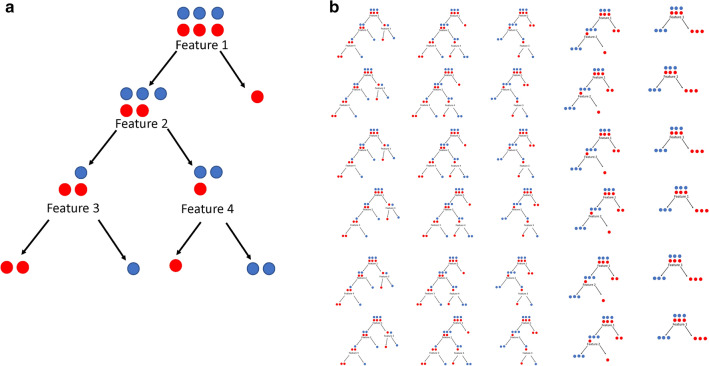
Classification and Regression Trees (CART) and Random Forest. **a** Diagram showing a single CART. CARTs take a heterogeneous group of data and repeatedly split on feature values to create more homogeneous groups. **b** Diagram showing a random forest. A random forest is a collection of CARTs, each running on a slightly different subset of the same data set

The next machine that will be discussed in this review is random forest (RF). RF is built upon the concept of the classification and regression tree (CART), which takes a group of heterogeneous data and repeatedly splits the original data set into more homogeneous groups (termed nodes) based on features (Breiman 2001; Malley et al. 2011) (Fig. 2a). Deciding which feature to initiate the split at a particular node is often determined by its increase in purity in the resulting nodes. Purity is the measure of class homogeneity at a particular node; for instance, the percentage of cases vs. controls. The higher the proportion of one class, the purer the node. There are multiple measures of purity, including the popular Gini index (Malley et al. 2011). Splitting ceases when a predetermined purity threshold is reached; nodes that are not split further are called terminal nodes. The subjects in the terminal nodes are tallied and a simple majority then determines node classification (Malley et al. 2011). RF is just a collection of CARTs (hence the name “forest”) that are built upon bootstrap samples of the original dataset. Classification is determined by a majority vote across the trees of the forest (Breiman 2001) (Fig. 2b). A genetics example using RF would be the use of classification trees to perform repeated splitting of case/control data by SNP genotypes to determine which alleles affect case classification. RF can be run as regression trees on continuous data, as well.

The inherently model-free approach has made RF a popular machine in genetic analysis. RF has been used in the prediction of variant pathogenicity (Ioannidis et al. 2016), association studies (Szymczak et al. 2009), RNA-seq and expression data (Guo et al. 2020), next-generation sequencing calling quality control (Li et al. 2019), and DNA methylation (Wilhelm 2014). RF is often used for purely predictive purposes; however, feature importance (often called variable importance in RF parlance) is relatively trivial to calculate in RF. Thus, there are more examples of methods to determine feature importance in RF than for other machines.

There are several different ways that feature importance can be calculated in RF. One method is the Gini importance. As mentioned above, at each node, features are assigned a Gini index (a measure of node purity) to determine which feature to perform the split. These Gini indices for features can be averaged across all nodes and trees to determine the Gini importance of the feature in the analysis. Gini importance has been shown to have biases, including toward higher frequency features (Nicodemus 2011), which would include bias toward more common SNPs in genetic studies (Boulesteix et al. 2012). A recent approach by Nembrini et al. (2018) has sought to remove these biases and has been shown to be powerful in genetic analysis.

Another major method of calculating feature importance in RF is permutation, where features are systematically permuted and their effect on prediction is observed (Malley et al. 2011). Importance scores can then be created for each feature, allowing for the ability to rank features by importance (Szymczak et al. 2016). The permutation approach lacks the biases of the Gini importance, but takes significantly longer to compute, as each feature needs to be permuted individually across all the trees in the forest while keeping the other features constant. It should be noted that permutation-based importance has biases, as well, including increased false positives due to unimportant features correlated with important features. Several conditional corrections have been proposed, including the permutation importance (PIMP) approach (Altmann et al. 2010) and the conditional permutation approach (Strobl et al. 2008). However, in genetic association studies, detection of association to groups of variants that are in strong LD with each other are not considered serious false positives, because they direct the researcher to the region of the genome that may harbor a causal variant. Random jungle (RJ) is a software package that can rapidly analyze GWAS data using RF (Schwarz et al. 2010). RJ can calculate feature importance based on both Gini importance and permutation-derived importance. It also includes a backwards elimination method, in which RFs are iteratively fitted, with features with low importance scores being removed at each step. RJ was used to identify new genes associated with Crohn’s disease (Schwarz et al. 2010). The initial RJ software has been subsumed and improved by the R package ranger, which is even more efficient at analyzing high-dimensional data (Wright and Ziegler 2017).

RF has become popular for predicting variant pathogenicity. One such example is the rare exome variant ensemble learner (REVEL). REVEL is a RF-based method that is used to predict the pathogenicity of rare missense variants (Ioannidis et al. 2016). REVEL was trained with recently discovered pathogenic and neutral variants and takes as input functional scores from well-known prediction programs like SIFT (Ng and Henikoff 2003) and FATHMM (Shihab et al. 2013) and conservation scores like GERP++ and SiPhy. Not only did REVEL have better overall performance than other methods, but it was able to identify which features were most important in REVEL’s pathogenicity predictions (Ioannidis et al. 2016). REVEL found that the functional scores from FATHMM (Shihab et al. 2013) and VEST (Carter et al. 2013) were the most important features in its pathogenicity predictions, and that functional scores in general were more important than conservation (Ioannidis et al. 2016). These feature importance scores give us valuable insight into what is going on within REVEL’s black box, as well as let us know that FATHMM and VEST may be more effective than other functional annotation programs of interest and that, perhaps, we should put greater weight on functional scores compared to conservation scores.

In a similar manner, feature importance allows us to peek inside the black box of Open Target Genetics’ prioritization machines. These prioritization machines are based on a gradient boosting classifier (not strictly a RF but uses CARTs like RF) (Ghoussaini et al. 2021). Locus-to-gene (L2G) is one such machine that incorporates different features like distance to gene, expression data, and chromatin interactions to prioritization causal genes from a number of SNPs from a significant locus (Ghoussaini et al. 2021). This is especially useful when looking at many noncoding SNPs, or SNPs along a linked or associated haplotype. While an L2G score is given for each gene, feature importance metrics show that distance to gene is more important than other factors like gene expression.

RF and other ML methods are used in phenotype definition, as well. While most genetic analyses focus primarily on genotypes, the characterization of phenotypes can be just as critical. ML approaches can be used to find disease subgroups that might not be identified through traditional analyses. This sort of clustering analysis is an example of unsupervised learning, where machines (such as RF) look for hidden patterns in the dataset (Shi and Horvath 2006). This is opposed to supervised ML, where machines are looking to accurately classify or predict (the subject of the majority of this manuscript).

Crucial to this effort is the analysis of electronic health records (EHRs), which contain copious amounts of diverse data on patients that can be mined to elucidate homogeneous subgroups from heterogeneous traits (Basile and Ritchie 2018). These analyses can be used to identify unique disease subsets through prediction or identify novel sets of features to better classify affected subjects through feature importance. For instance, Teixeira et al. used numerous ML techniques to analyze EHRs to identify individuals with hypertension, with RF being the most effective (Teixeira et al. 2017). Looking at feature importance determined that blood pressure measurements, which is traditionally used to diagnose clinical hypertension, was the worst-performing feature at predicting hypertension. Other EHR information, such as vitals and medications, was much more effective (Teixeira et al. 2017).

The detection of epistatic interactions between variants is another popular area of active research in RF feature importance, even in high-dimensional data (Lunetta et al. 2004; Winham et al. 2013). RF is well suited to identify interactions under the theory that if variants are indeed interacting, then it is likely that once one variant is chosen as a splitting criterion, the interacting variant may shortly follow. This will rank both features as significant and also provides a mechanism to identify higher order interactions (Holzinger et al. 2016). While there are multiple ways to determine RF feature importance in epistasis, Orlenko and Moore determined that permutation-derived importance metrics are more precise at identifying interactions (Orlenko and Moore 2021).

Various flavors of RF have been developed to detect epistatic interactions amongst variants. For instance, permuted random forest (pRF) identifies interacting SNP pairs by systematically permuting interactions between a pair of SNPs and determining which SNP pairs cause the greatest reduction in prediction power (Li et al. 2016). Random forest fishing (RFF) is an iterative approach that has been shown to identify important variants even when no main effects are present on the variants (Yang and Charles Gu 2014).

Sliding window sequential forward feature selection (SWSFS) uses SNP genotypes as categorical features and uses a sliding window approach to select a small number of candidate SNPs that minimized classification error using Gini importance, as opposed to permutation-derived importance (Jiang et al. 2009). SWSFS was used to test up to three-way interactions and was successfully used to identify SNPs associated with age-related macular degeneration.

Recurrent relative variable importance measure (r2VIM) adds the principle of recurrency to RF to identify epistatic interactions in SNP genotype data (Szymczak et al. 2016). In a single run of RF, false positives may have higher importance scores than true predictors simply by chance. Recurrency eliminates this problem by running multiple independent RF analyses on the same data set, using a different starting seed. Permutation-based importance scores are then calculated for each feature for each of the analyses, which are termed relative importance scores. The median of these relative importance scores is then taken to represent the true importance score. This serves to reduce false positives while keeping true predictors with large importance scores. r2VIM was shown to control false-positive rates and identify main effect SNPs (Szymczak et al. 2016). It has also been shown to identify epistatically interacting SNPs as important, even when these SNPs have no main effects on the trait (Holzinger et al. 2015).

One might wonder with multiple ways to calculate feature importance in RF, is one method more effective than the other? Degenhardt et al. undertook a comparison of feature selection methods, including standard permutation-derived approaches and the recurrent approach of r2VIM (Degenhardt et al. 2019). Other evaluated approaches included that of Boruta, which calculates importance scores by creating shadow features (shadow variables) by doubling each feature and permuting it. The importance scores of the real features are then compared to that the shadow features (Kursa and Rudnicki 2010). A fourth method was that of Vita, which involves dividing the overall data into two equal, independent subsets and estimating variable importance using the other set; an importance score called the hold-out importance is calculated (Janitza et al. 2018). After comparison, Degenhardt et al. concluded that Boruta and Vita were the most powerful approaches in simulation studies using high-dimensional data, noting that Vita is significantly faster (Degenhardt et al. 2019).

### Artificial neural networks

**Fig. 3:**
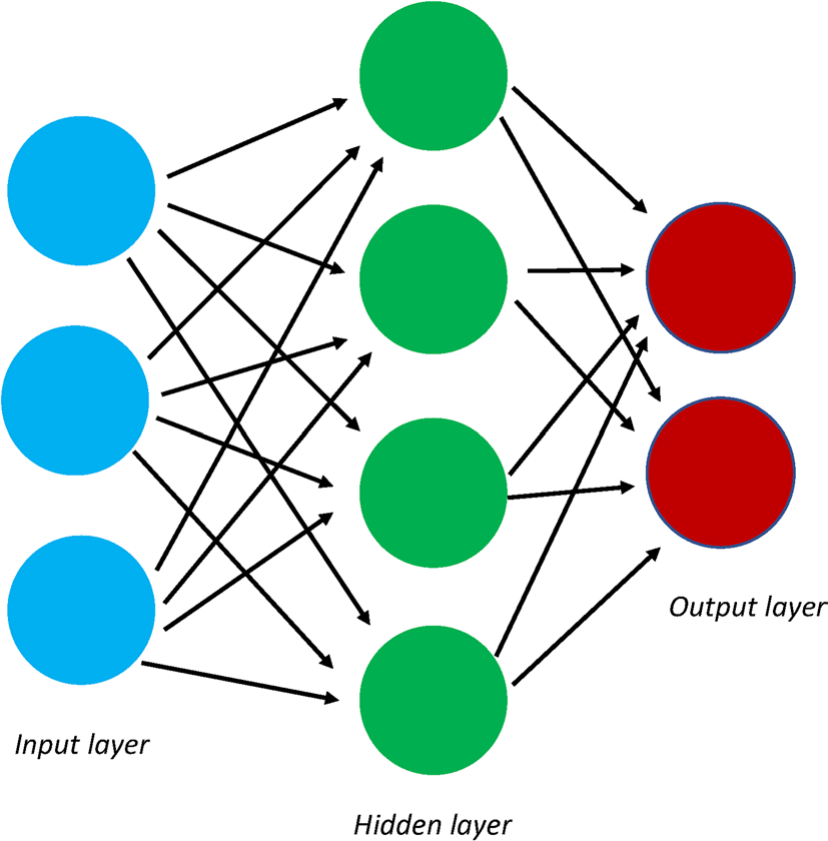
Artificial neural networks. A schematic of an artificial neural network. Data are analyzed by different models, the results of which are passed onto a new set of models. In this example, data are first analyzed in the input layer (blue). The results are then passed onto an intermediate layer, called a hidden layer (green). Finally, the results of the hidden layer are passed onto and analyzed by the models of the output layer (red)

Artificial neural networks (ANNs), also known as neural networks (NNs), are a more complex type of machine. They consist of multiple small models (nodes) linked together, feeding the output of one model into the input of another. In this way, they loosely resemble the neurons and synapses of the brain as information is rapidly transmitted from one model to another. ANNs take input data that are given to an initial set of small models (which can just be simple models like linear/logistic regression). The output from the first model is then transmitted to a second set of models as a weighted sum. This process is then repeated as multiple small models are combined into distinct weighted sums until a final output is reached. The intermediate sums, which are not reported, are referred to as the hidden layers (Malley et al. 2011). Complex mechanisms such as backpropagation, where information is passed in the reverse direction to better fit the network, and gradient descent, an optimization algorithm used to minimize predictive loss, are used in this process. Traditional ANNs rarely have more than one or two hidden layers (Fig. 3). Furthermore, due to the often sparse signals in genetic data, fully connected ANNs (meaning each “neuron” in one layer feeds its data into every other “neuron” in the succeeding layer) are not always used. Instead, convolutional layers are used, where “neurons” are only connected some of the “neurons” in the succeeding layer.

Due to their model-free assumptions and their ability to interpret a wide range of data types, ANNs have been frequently used in genetics. They are especially popular prediction tools, whether it be for protein structure and folding (Cai et al. 2003), variant genotype calling in microarrays and sequencers (Poplin et al. 2018), or the question of whether tumors will metastasize (Wang and Yu 2020).

ANNs can be used for feature importance, as well, though they are more commonly used for prediction and are more of a black box than other methods. Olden and Jackson proposed a randomization approach for ANNs that allows users to quantitatively assess both the individual and interactive effects of the input variables in the network prediction process, as well as evaluate the overall contributions of the variables to the prediction, which they demonstrated effectively using ecological examples (Olden and Jackson 2002). Initially, in genetics, ANNs were used as an alternative to traditional single locus and multilocus association methods, meaning that ANNs would evaluate the potential association of a single marker on disease status. Curtis found their power comparable or better to other traditional methods like haplotype analysis (Curtis 2007), but there has not been much movement in this aspect for over a decade, likely due to the effectiveness of traditional methods at finding main effects in association studies.

However, this does not mean that feature importance using ANNs in genetics has remained stagnant. ANNs have been particularly useful in gene expression and gene–gene interaction studies. Tong and Schierz (2011) developed a hybrid ANN algorithm for gene expression data that specifically detects genes that are good predictors, called genetic analysis neural network (GANN). The ANN algorithm is combined with a genetic programming (GP) algorithm. GP algorithms are inspired by evolution; they generate a population of solutions which proceed to mutate and recombine over a preset number of generations. The best solutions are determined via a fitness metric. GANN emphasizes importance of features, and uses the ANN part of the algorithm to determine the fitness function of the GA. Results using expression data found that GANN was able identify genes that had been identified as significant using the traditional methods as well as novel genes that were biologically relevant to the trait being studied (Tong and Schierz 2011). The significant genes from the initial GANN study were later used to develop the artificial neural network inference (ANNI) approach to identify epistatic interactions. This procedure used ANNI to explore all potential influences of genes amongst themselves (Tong et al. 2014). A matrix of interactions that can be ranked by value is the output.

The Analysis Tool for Heritability and Environmental Network Associations (ATHENA) uses an ANN algorithm to perform a suite of analyses using multiple types of input data, including microarray, sequence, and expression data (Holzinger et al. 2014). ATHENA is designed to test pairwise interactions between variants and uses a modified ANN called a grammatical evolution neural network (GENN). GENNs transcribe input data, such as SNP genotypes, into an internal grammar to increase efficiency (Turner et al. 2010). They proceed in a manner similar to GP, evolving the heterogeneous mix of weights and inputs that undergo mating crossovers and recombinations that test two-SNP models (Holzinger et al. 2014). Fitness is recorded for each model and models with the highest fitness are selected for crossover and reproduction; this is done for preset number of generations. ATHENA uses additional biological information to create its two-SNP interaction models, including pathway information from KEGG and functional information from Gene Ontology (Holzinger et al. 2014). ATHENA’s output includes all features from the best model as well as their cross-validation scores; letting users observe which features have informed the best model.

### Deep learning

**Fig. 4:**
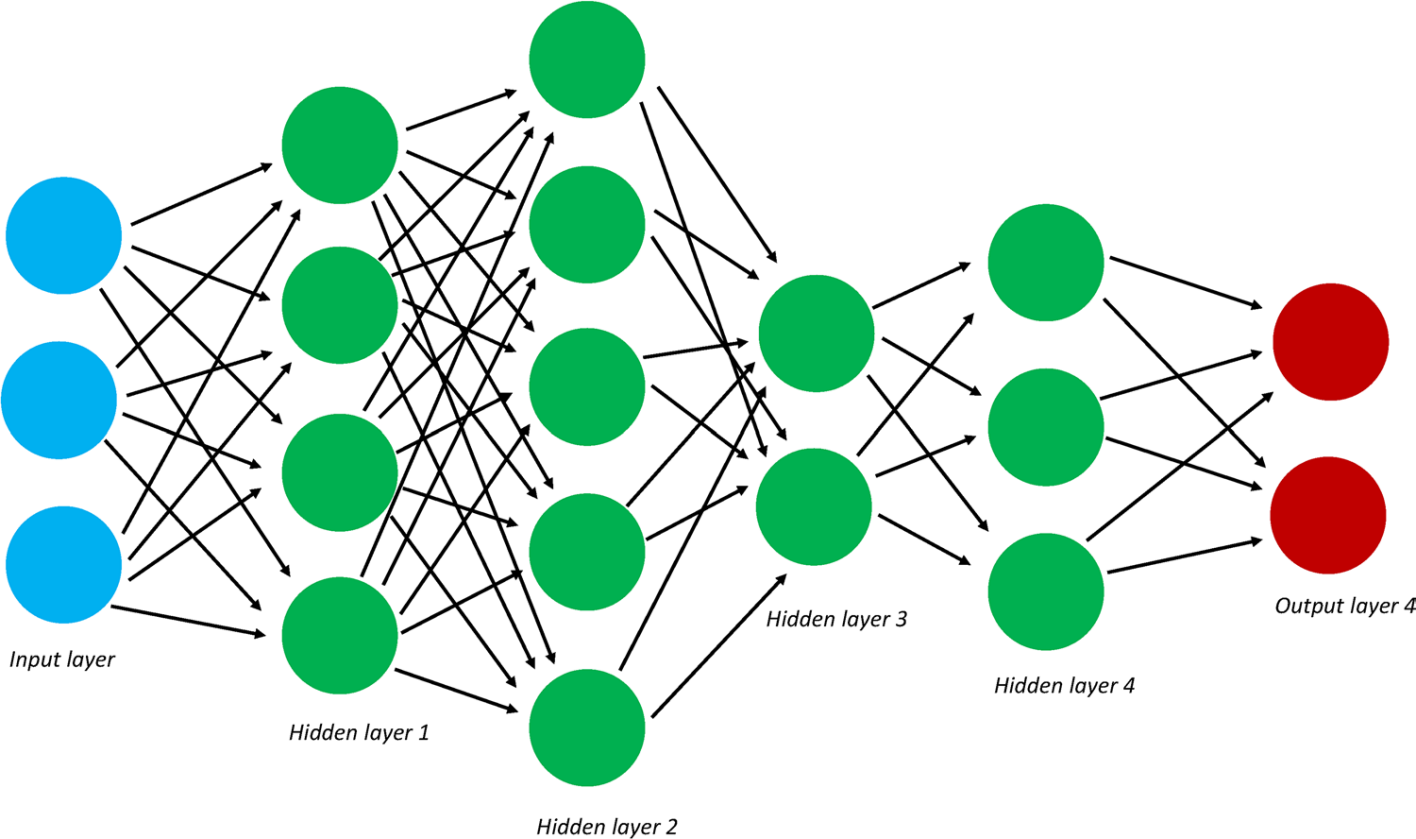
Deep learning. A schematic of a deep learning machine. Deep learning is a specialized version of artificial neural networks that contain many additional hidden layers

A specialized type of ANN that has gained popularity in recent years is called deep learning. Deep learning relies upon deep neural networks, which follow the same principle as ANNs. Initial models compute some result from input and the output from the initial model is transmitted to another model. The process is repeated, producing more complex outputs along the way. However, the deep neural networks that underpin deep learning contain many hidden layers (as opposed to just one or two in ANNs) (Fig. 4). Recall the hidden layers are the intermediate models between the first input model and the final output model that is reported. Deep learning also can contain far more complex architecture than simple multilayered ANNs, including convolutional or recurrent layers. Deep learning has exploded in a variety of fields within recent years due to its ability to handle extremely complex, heterogeneous data (including image data), its model free assumptions, and its relative ease of use for non-experts.

In genetics, deep learning has been applied to DNA sequence data to predict the effect of noncoding variants (Zhou et al. 2018; Zhou and Troyanskaya 2015), pathogenicity of exonic variants (Sundaram et al. 2018), and the identification of regulatory motifs (Kelley et al. 2018) and transcription factor-binding sites (Wang et al. 2018). Given their proliferation in both biology and genetics in particular in the past 5 years, a full review of deep learning in genetics is well beyond the scope of this paper, but the interested reader is directed to an excellent review by Eraslan et al. (2019).

The vast majority of deep learning applications in genetics is concerned with prediction. It is possible to estimate feature importance, but Eraslan et al. noted that benchmarking and thresholding of importance scores in genomic data have not been well tested and should be compared to known simulated data (Eraslan et al. 2019). Feature importance in deep learning is calculated either by perturbation (changing a value and recording the subsequent change in prediction) or by backpropagation through the network (Eraslan et al. 2019).

Many of the pathogenicity and regulatory prediction programs have some sort of importance metric to determine which features affect their models. For instance, the regulatory sequence prediction program Basset (Kelley et al. 2016) uses perturbation of stretches of DNA to determine which sequence motifs are important to predicting regulatory sequences. DeepSEA (Zhou and Troyanskaya 2015), which predicts the effect of noncoding variants, uses a similar perturbation approach by changing single nucleotides of sequence data. DeepSEA then provides a functional significance score for noncoding variants based on chromatin effect predictions and evolutionary conservation. Regulatory predictions from DeepSEA have recently been integrated into a new program called DeepWAS (Arloth et al. 2020). While most functional annotation of GWAS is performed post hoc, DeepWAS combines the association analysis and functional annotation from DeepSEA in a single step to identify disease-associated loci that are likely to be regulatory variants. DeepWAS was successfully tested on real data, including multiple sclerosis and height.

Deep Feature Interaction Maps (DFIM) (Greenside et al. 2018) is a deep learning machine created to identify epistatic interactions between sequences of the genome. DFIM computes a novel Feature Interaction Score (FIS) between a target sequence and source sequence by systematically perturbing nucleotides in the source feature. A computationally efficient backpropagation is then used to calculate FIS between all pairs of nucleotides or regulatory motifs in a DNA sequence, determining which changes have the greatest effect. DFIM was used to identify synergistic interactions GATA1 and TAL1 motifs as well as other regulatory genetic variants (Greenside et al. 2018).

PrimateAI, a deep neural network that is trained on using the primary amino acid sequences, uses variants from non-human primates like chimpanzees, gorillas, and orangutans to better predict classification of pathogenic variants in humans (Sundaram et al. 2018). Using a deep neural network built to extract features from just the primary amino acid sequence of the variant of interest and flanking variants, PrimateAI was able to predict variant pathogenicity at a higher percentage than other prediction programs and identify novel candidate genes for intellectual disability. While the goal of PrimateAI was classification (they successfully estimated pathogenicity on over 70 million variants), the algorithm does look at effects of features on its neural network. For instance, it was found that each of six non-human primate genomes increased prediction accuracy while adding non-primate mammalian genomes (e.g., pig or cow) decreased accuracy (Sundaram et al. 2018). This led the authors to note that additional sequencing of non-human primates will increase pathogenicity prediction in humans. This is a nice example of how opening up the black box of these complex deep neural networks and looking at the actual effect of features on prediction leads to valuable feedback about how to improve the overall machine.

### Support vector machines

**Fig. 5:**
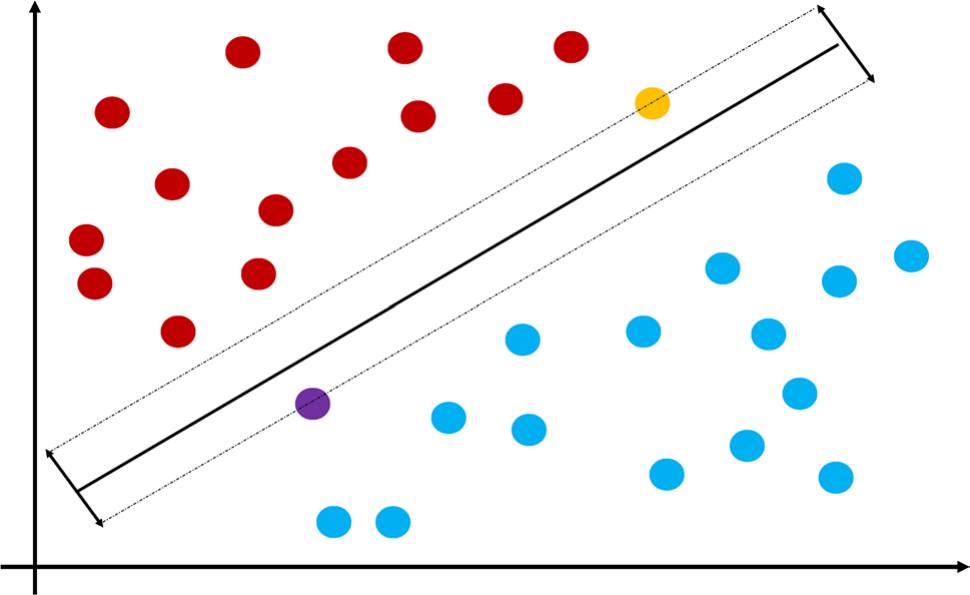
Support vector machines. A diagram showing an example of a support vector machine. Subjects are plotted based on feature values, and a special boundary called the hyperplane is formed to classify individuals. The hyperplane is oriented as far as possible from the two closest individuals in each class (in this example, the orange and purple individuals)

Support vector machines (SVMs) are a large class of ML algorithms that have become popular in part because of their mathematical elegance and their ability to handle large numbers of features (Malley et al. 2011). SVMs work this way—within a set of data to be classified, there exists a decision boundary that can be drawn through the data to enable classification. SVMs orient this boundary, which is called the hyperplane, so that it is as far as possible from the two closest points of each class (Huang et al. 2018) (Fig. 5). A kernel function is used in higher dimensional models to calculate the hyperplane more efficiently. Essentially, the kernel allows for datapoints in multiple dimensions to be treated as linear data, thus easily computing the distance between datapoints. Without the kernel function, this would be much more difficult. There are several different types of kernel functions, which are expounded on in the excellent text by Schölkopf et al. (2003). Popular kernels for SVMs include linear kernels, radial basis function (RBF) kernels for non-linear data, as well as Gaussian and polynomial kernels. One particular kernel of note for genetic data is the string kernel, which takes as input (long or short) sequences of text (called strings in programming). This is very useful in DNA-seq analysis where long stretches of genotypes can be compared. SVMs can classify continuous data, as well; in these cases, the SVM is usually referred to as support vector regression (SVR).

As they are powerful classifiers, SVMs are used for prediction in a variety of genetic scenarios. For instance, SVMs have been trained to use RNA-seq data to identify patients with thyroid cancer (Shen et al. 2020), proteomic data to identify different breast cancer subtypes (Tyanova et al. 2016), sequence data to detect somatic tumor mutations (Mao et al. 2021), and cell-free DNA to diagnose cancer (Liu et al. 2021).

One of the best known SVMs in genetics studies is Combined Annotation-Dependent Depletion (CADD) (Kircher et al. 2014; Rentzsch et al. 2019), a popular annotation program for identifying variant pathogenicity. CADD draws from a variety of different features including allelic diversity, annotations of functionality, pathogenicity, disease severity, and experimentally measured regulatory effects to create a quantitative CADD score, which can be used to prioritize deleterious exonic variants.

Though they are primarily used as predictors, SVMs offer feature importance metrics as well. One notable SVM is the support vector recursive feature elimination (SVM-RFE) (Guyon et al. 2002). SVM-RFE first runs on all features and applies an importance score to each feature that is based on how well each feature classifies the training data. Lowest scores are iteratively removed, and the algorithm stops when all features are important. Guyon et al. first successfully used this method to select genes for cancer prediction (Guyon et al. 2002).

Hu et al. (2016) used SVM-RFE in an algorithm containing SVM in combination with random forest (RF) to identify genes responsible for cell differentiation. Using single-cell RNA-seq data from neocortical cells and neural progenitor cells, the SVM-RFE/RF classifiers were able to identify 38 genes that best predicted the differentiation of neocortical cells from the neural progenitor cells. Similarly, Xu et al. (2017) were able to use SVMs trained on colon cancer microarray expression data to identify 15 genes as predictors of recurrence risk and prognosis in colon cancer patients.

SVMs have been also used for SNP selection, as well. De Oliveira et al. (2014) used support vector regression to identify SNPs that were associated with a simulated phenotype. The method was able to identify at least some of the causal SNPs, even in the case of polygenic and epistatic effects. The method was also particularly effective at reducing noise variants to yield a smaller set of variants.

MSIpred is an SVM algorithm constructed to detect microsatellite instability (MSI), a condition associated with a high degree of polymorphisms associated with several types of tumors (Wang and Liang 2018). MSIpred uses mutational load data created from whole-exome data.  While MSIpred’s particular goal is classification, a RF process can be used to determine which features are important.

### Not so different after all

One of the important things to note about the methods that were described above is that though they may seem quite different, multiple connections can be drawn between these seemingly disparate algorithms. They are indeed working to solve similar problems. For example, both the SVM kernel functions and deep learning try to tackle the problem of high-dimensional data by reducing the dimensionality to a more palatable linear problem. Obviously, this is done in different ways—SVM kernels embed the data in a space of infinite dimensions where linear separation can be performed and deep learning “learned” to approximate a linear boundary—but the same problem is being solved in both cases. Further RF can be thought of as an adaptive *k*-NN (Lin and Jeon 2006) by considering the distance between data points as the proportion of shared terminal nodes. In this sense, the terminal node essentially becomes the “neighborhood” seen in *k*-NNs. This shows how not only these methods are approaching the similar problems, but also the interconnectedness of many of these algorithms.

### Machine selection and parameter tuning

The vast number of ML methods can seem overwhelming, especially to geneticists that are not experts in ML. It is difficult to know which method might be appropriate for a given data set. Furthermore, many ML algorithms require further input than just genetic data, including parameters that need to be tuned and optimized, often by a trial-and-error approach, such as the k term in k-NN. Again, this can be a daunting task to ML non-experts.

One strategy is to run multiple different machines on the same data and then incorporate that data into a new machine. This is the theory behind methods such as synthetic random forest (Ishwaran and Malley 2014) quantitative (regression), the similarity-binning averaging approach (Bella et al. 2013), and optimal crowd (Battogtokh et al. 2017). Synthetic random forests (SRFs) operate under the assumption that differing data might be better fit by a different number of terminal nodes; though tuning this by hand is not feasible. SRFs work by running multiple RFs of varying terminal node size and calculating the predicted value of each RF (the synthetic feature). The synthetic features are then placed into a new RF with the original features (the SRF); SRFs outperform the traditional RFs and optimized RFs (Ishwaran and Malley 2014). The similarity-binning average approach looks to calibrate a model by first running a given model and obtaining the estimated probabilities associated with each dataset, with the estimated probabilities combined with each instance creating a new dataset. The model is then run a second time, and the probabilities from the second run of the model are then placed with the most similar instances (usually determined by *k*-NN) from the first run, creating a bin; with the probability of classification just being the average of the bins. This method has been shown to be empirically better than other calibration methods (Bella et al. 2013). Optimal crowd takes predictions from a family of machines (like RFs and SVMs) that analyzed the same binary data. Using these multiple predictions on the same data allows the machines in the optimal crowd to learn from each other and make a new classification. Optimal crowd has been shown to be at least as good as the best machine in the family (Battogtokh et al. 2017). None of these methods offer feature importance metrics currently, however. We note that there is no one machine that is best for all datasets and problems. Thus, the choice of machines is very difficult, since there is no best machine for all circumstances. This dilemma is indeed the motivating factor for many autoML approaches described below.

Another approach is automated machine learning (autoML). This is a relatively new field that seeks to automate the parameter selection processes, taking the burden off the user. Thus, instead of requiring the analyst to tune parameters or models, autoML essentially does this heavy lifting for you by building an ML pipeline that optimizes models by model selection, parameter tuning, etc. (Le et al. 2020). Most autoML models focus primarily on prediction, but Tree-based Pipeline Optimization (TPOT), a GP-based autoML method does generate permutation-derived feature importance scores (Orlenko et al. 2020). TPOT has been tested on genetic data, including the evaluation biomarkers for the prediction of heart disease (Chirinos et al. 2020). Currently, autoML methods take a lot of computational power to run, and thus have been confined to relatively small sets of features (Le et al. 2018, 2020). Recent developments in TPOT have increased scalability in large data sets, including a feature selection set that allows for the specification of features into subsets (Le et al. 2020). TPOT also now allows for covariate adjustment, which drastically improved feature importance scores by eliminating false positives in a gene expression study (Manduchi et al. 2020).

## Conclusion

Machine learning approaches have greatly increased our ability in genetics to analyze complex data sets, as well as ask more intricate questions. ML approaches in genetics have been mostly commonly used to elicit good predictions, for instance to identify cancer patients through RNA-seq (Shen et al. 2020). However, in genetics feature importance is also a critical field. Often, it is just as important to know what makes a good prediction as it is to get a good prediction by blindly feeding data into a black box. Feature importance allows for ranking of particular features, whether it be for identifying epistatic interaction (Holzinger et al. 2015; Szymczak et al. 2016) or simply determining which features used by a machine are responsible for the best prediction (Ioannidis et al. 2016; Sundaram et al. 2018). While we have primarily discussed feature importance as a way of identifying features that most affect a prediction, we note that importance metrics could be used in the reverse way, meaning that features that are deemed important for poorly performing classifiers might themselves be less informative.

As noted in this review, there are numerous genetic programs that give feature importance scores that can answer a variety of questions, including variant pathogenicity and epistatic interactions. Many of these programs are built using variants of popular, well-known machines like random forest, artificial neural networks, deep learning, and support vector machines. Newer methods, like the autoML approaches (Le et al. 2020), decrease the expertise needed to run some of these complex machines, by tuning parameters automatically instead of requiring user input (though interpretation of results may still require an understanding of the machine). It is clear that genetic data are only to increase in complexity and quantity, so the importance of novel ML approaches will only increase in the coming years. Feature importance metrics for these methods will be critical, as it allows for the researcher to not only identify important variables for prediction, but to see what is happening within the black box of many of these algorithms.

## Data Availability

This is a review article, so there are no original data associated with it.

## Code availability

This is a review article, so there is no unique code associated with this work.
